# The Effectiveness of Active Screen Method in Ion Nitriding Grade 5 Titanium Alloy

**DOI:** 10.3390/ma14143951

**Published:** 2021-07-15

**Authors:** Tadeusz Frączek, Rafał Prusak, Marzena Ogórek, Zbigniew Skuza

**Affiliations:** 1 Department of Materials Engineering, Faculty of Production Engineering and Materials Technology, Czestochowa University of Technology, 42-201 Częstochowa, Poland; tadeusz.fraczek@pcz.pl; 2 Department of Production Management, Faculty of Production Engineering and Materials Technology, Czestochowa University of Technology, 42-201 Częstochowa, Poland; rafal.prusak@pcz.pl (R.P.); zbigniew.skuza@pcz.pl (Z.S.)

**Keywords:** titanium alloys, ion nitriding, active screen method, microhardness, microstructures

## Abstract

The study assessed the effect of ion nitriding on the properties of the surface layer of Grade 5 titanium alloy used, among others, in medicine. Titanium and its alloys have low hardness and insufficient wear resistance in conditions of friction which limits the use of these materials. The improvement of these properties is only possible by the appropriate modification of the surface layer of these alloys. The ion nitriding process was carried out in a wide temperature range, i.e., 530–590 °C, and in the time range 5–17 h. Two variants of nitriding were applied: cathodic (conventional) nitriding and nitriding using the active screen method. The research results presented in this article allow for stating that each of the applied nitriding variants improves the analysed properties (nitrogen diffusion depth, hardness, wear resistance, microstructure analysis and surface topography) of the surface layers in relation to the material before nitriding. The hardness increased in every nitriding variant (the use of the additional active screen increased the hardness to 1021 HK0.025). The greatest increase in titanium abrasion resistance was found for surfaces after cathodic nitriding with an active screen. Each of the applied nitriding variants resulted in surface development.

## 1. Introduction

Titanium and its alloys demonstrate good strength and corrosion resistance, and are also characterised by biocompatibility and low weight density [1]. The most popular processes among the processes aimed at improving the surface properties of Ti alloys include nitriding, oxidation and carburisation [2], with ion nitriding being the most common method of improving the surface properties of titanium and its alloys [1]. Titanium nitrides have good tribomechanical and biomedical properties. They are used to harden and protect cutting and sliding surfaces for industrial purposes and as a non-toxic outer surface for biomedical applications [3].

The influence of various surface treatment processes on the properties of titanium and its alloys is the subject of research by several authors. Balasubramanian et al. [1], while investigating the influence of ion nitriding on the mechanical, corrosive and abrasive properties of Grade 5 alloy (in an atmosphere of 80% N_2_ and 20% H_2_, at a temperature of 500 °C for 8 h), showed the formation of nitrides in the surface layer and an increase in corrosion resistance, wear resistance and hardness. Fouquet et al. [4] while using gas mixtures of N_2_-H_2_ at a pressure below 10 Pa at different process times and the temperature range of 500–900 °C, showed that, in a Grade 5 alloy, the glow discharge plasma enhances the formation of richer compounds, such as δ-TiN and ε-Ti_2_N nitrides, on the surface at a lower temperature than that obtained with the classical heat treatment. In addition, with longer process times, the ion treatment allows for the formation of a δ-TiN layer and below, the previously formed ε-Ti_2_N grains undergo nitrogen rearrangement with the remaining α-Ti grains, which leads to the formation of the α-TiN_0.26_ phase. While conducting the process in a gas mixture of 25% Ar-75% N_2_ at the process time of 1–4 h and at temperatures of 650–750 °C, the formation of δ-TiN and tetragonal ε-Ti_2_N phases in the modified layer was confirmed and it was observed that the surface hardness and abrasion resistance increases along with an increase in processing time and temperature [5]. Research by Mubarak et al. [6] showed that ion nitriding reduced the tangential force factor of Grade 5, and that the samples nitrided in the environment of nitrogen-hydrogen mixtures demonstrated higher hardness and lower tangential force factor compared to the samples nitrided in pure nitrogen.

Tests conducted at 850 and 900 °C revealed a complex multilayer microstructure resulting from diffusion on the surface [7]. The layers formed on the Ti-6Al-4V alloy were identified as δ-TiN phases (NaCl face-centred cubic type), δ′-Ti_2_N (central tetragonal), ε-Ti_2_N (tetragonal primitive), α (N) solid solution containing up to 19 at.% of nitrogen as well as Ti_2_AlN and other minor ternary Ti-Al-N phases. The topography of the nitrided layer of the alloy, examined under an atomic force microscope, showed a clear surface development. The relationship between the microstructure of the alloys and their micromechanical and tribological properties was determined. The improved properties were related to the presence of nanocrystalline δ-TiN in the outermost sublayer on top of the δ′ + ε-Ti_2_N and Ti_2_AlN [7] crystal layers. The study of the microstructure and phase composition of the Ti6Al4V alloy DC PN and AS PN treated at temperatures of 680 °C and 740 °C showed that the transition from DC PN to AS PN resulted in covering it with a dense layer of TiN reinforced with martensite α ″ -Ti (N) and layers of the Ti_3_Al type, i.e., the same as in the previous case (DC PN) in the absence of the layer δ. In addition, the mechanism of nitriding changed from absorption-diffusion of nitrogen ions to adsorption-diffusion of these ions, which was the cause of slowing down the nucleation rate and growth of both the compound and diffusion layers [8]. Higher levels of ionisation obtained using the DC triode configuration allow for shorter treatment time and lower nitriding temperature required to obtain hard and sufficiently deep nitrided layers in the Ti6Al4V alloy [9]. The surface hardness of 560–600 HV0.05 and thickness of the nitrided layer of 30–40 μm were obtained at the temperature of 700 °C. As revealed by tests, up to this nitriding temperature, hardening is performed mainly by nitrogen diffusion into the α-Ti phase [9]. Tests into the influence of low-temperature nitriding on the fatigue properties of Ti-6Al-4V titanium alloy [10] allowed for stating that the nitrogen compound layer was formed on the nitrided surface at temperatures higher than 600 °C, while nitrogen diffusion was observed on the nitrided surface at 550 °C. The fatigue strength for Ti-6Al-4V tended to increase as the nitriding temperature decreased. Nitriding at 550 °C increased the fatigue strength of Ti-6Al-4V due to the formation of a surface without a layer of nitrogen compound and reduced grain coarseness [10].

Tests conducted on samples of industrially pure titanium, at various process temperatures (700–1020 °C) and at a pressure of 5 Pa, showed that, with increasing temperature, the nitrogen content, the depth of the nitride layer, microhardness and wear resistance increase [11]. In other tests (with the process parameters: temp. 700 °C and 750 °C, times 2 and 4 h in NH_3_ and N_2_ + NH_3_ gas mixture in ratio 2:1) showed that ion nitriding of industrially pure titanium at a temperature of 750 °C for 4 h significantly improved its wear resistance, lowered the friction coefficient and increased corrosion resistance due to the formation of a thicker Ti-N layer [12]. Kapczinski et al. [13] showed that an improvement in tribological properties was obtained for the treated samples with the process duration of 3 h; in N_2_-H_2_ plasma atmosphere; at nitriding temperatures of 600 and 800 °C. The carried out tests [14] also allowed for showing that the wear resistance of titanium tested under vacuum conditions is increased due to the formation of a hard nitrided layer. At the same time the surface roughness, thickness and hardness of the nitrided layer increased with an increase in the nitriding temperature.

Nitriding high-alloy Ti_5_Al_4_V_2_Mo titanium material at temperatures of 500–900 °C in an Ar-N_2_ mixture at a pressure of 300–450 Pa for 3–4 h allowed for stating that the hardness on the surface increases with temperature, and at 900 °C, it is 1.7 times greater than the value of the original sample. At the same time, the hardness decreased with the depth from the surface [15]. Zhechev et al. [16] while examining various titanium alloys and using different process times and temperatures, found that the microstructure of nitrided alloys at temperatures below their β transition temperatures is homogeneous, and that with an increase in temperature above the β transition temperatures, the microstructure changes into an irregular one. At the same time, it was confirmed that the microhardness increased with an increase in nitriding temperature and time.

Ion processes allow for the formation of surface layers with a specific structure, phase, and chemical composition, which enable significant improvement in the tribological properties of titanium alloys. In this paper, the effectiveness of ion nitriding by the active screen method of Grade 5 titanium alloy was evaluated.

This alloy has been commonly used in medicine since the 1980s; however, low wear resistance under friction conditions limits its application. Improvement of performance characteristics is possible through the appropriate modification of the surface layer of this alloy through surface engineering methods. An analysis of literature data regarding surface engineering methods and obtained properties of the surface layer indicates that the introduction of plasma into treatment processes creates conditions for increasing the effectiveness of these processes, as well as improving the properties of produced surface layers [17,18]. One of the frequently used surface treatment methods is the glow discharge nitriding process. It was found in numerous papers that the nitrided layers formed in these conditions meet the high requirements in terms of corrosion resistance and wear resistance under friction conditions.

The comparison of nitriding technologies allows for stating that conventional ion nitriding—the processed element constitutes the cathode—is an imperfect solution, taking the technology criterion into account. The application of an active screen is an innovative solution which intensifies the process and improves its effectiveness, as well as performance characteristics of the formed nitrided layers [19,20]. It should be mentioned that the ion nitriding technology is generally not applied in industrial practice, and its application would bring tangible economic benefits.

## 2. Materials and Methods

The research material for carrying out the ion nitriding processes and assessing the effectiveness of these processes was the two-phase α + β Grade 5 titanium alloy, with the chemical composition compliant with the certificate issued by Daido Steel Co., Ltd. (Osaka, Japan) listed in Table 1. This alloy is widely used in the aerospace, chemical and medical industries.

**Figure 1:**
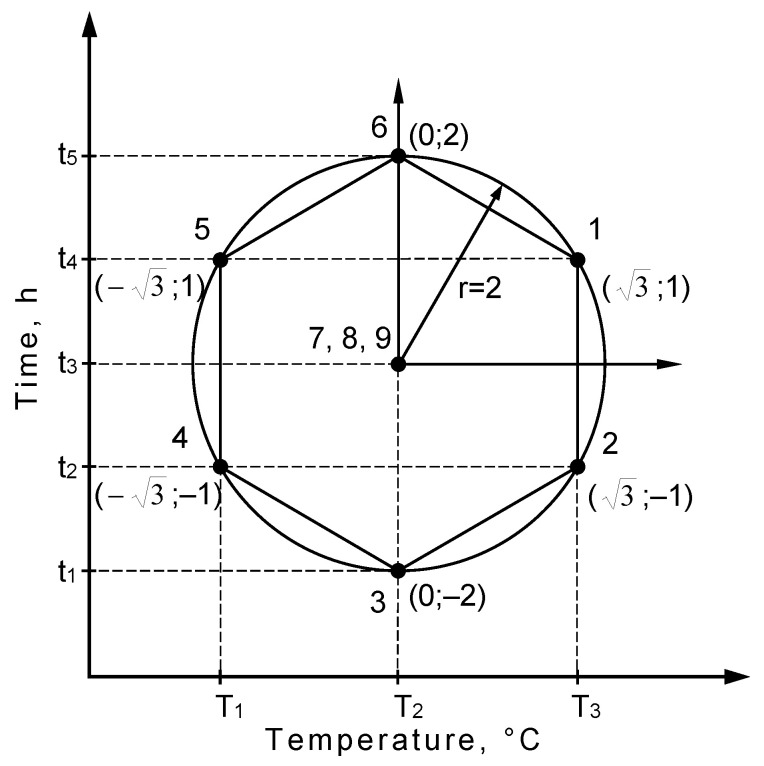
Diagram of planning the conditions of the nitriding process.

The ion nitriding process was carried out in a glow chamber with a cooled anode. The nitriding process was carried out in accordance with the adopted rotatable model of mathematical experiment planning (Figure 1).

Rotatable plans assume a constant value of the model inaccuracy for all points lying in the space of input quantities. The measure of a point location in the space is radius r of a sphere concentric with the examined factors’ axle arrangement. Moreover, the appropriate number of measurements in the program centre must be maintained [21].

**Figure 2:**
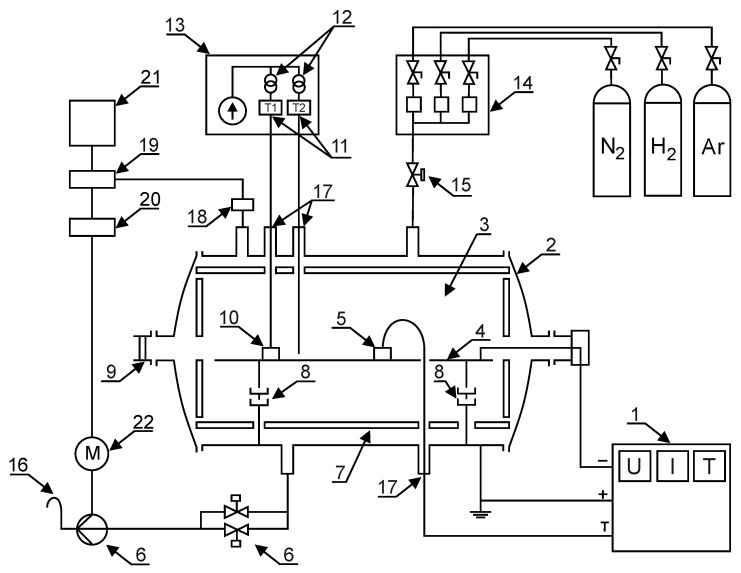
The schematic diagram of the glow discharge nitriding device: 1—MSS10 power supply, 2—water-cooled anode, 3—glow zone, 4—cathode, 5—temperature measurement sensors, 6—valve assembly and vacuum pump, 7—insulating screen, 8—insulators, 9—sight glass, 10—machined elements, 11—thermocouples, 12—transformers, 13—insulating casing of the temperature measuring system, 14—mass flow meters (gas dosing system), 15—electrovalve, 16—gas outlet, 17—insulation bushings, 18—measuring head, 19—vacuum gauge, 20—inverter, 21—computer, 22—rotary vacuum pump motor.

**Figure 3:**
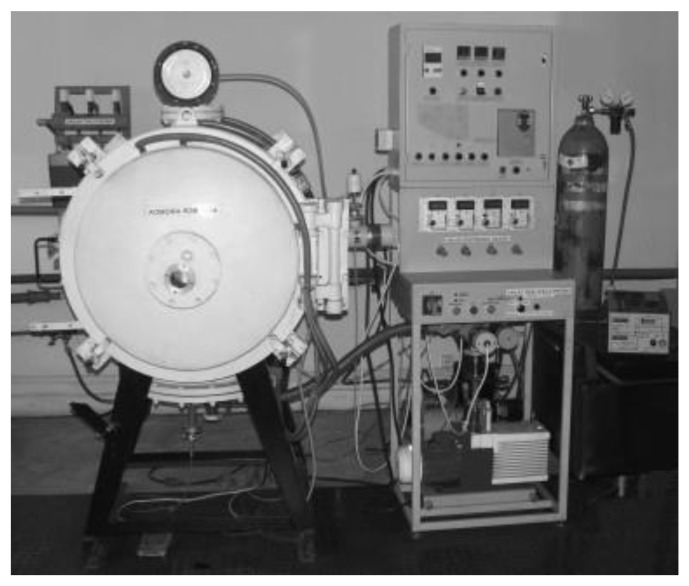
View of the fluorescent nitriding device used in the research.

The process of cathode glow discharge nitriding is well characterised in many studies and scientific publications [4,5,6,7,8,9,10,11,12,13,14]. The diagram of the analysis of the ion nitriding device is shown in Figure 2. Figure 3 shows the device for ion nitriding used in the research.

On the basis of the conducted preliminary tests, the conditions for the glow discharge nitriding processes of Grade 5 titanium alloy were determined (Table 2) [22,23,24,25].

**Figure 4:**
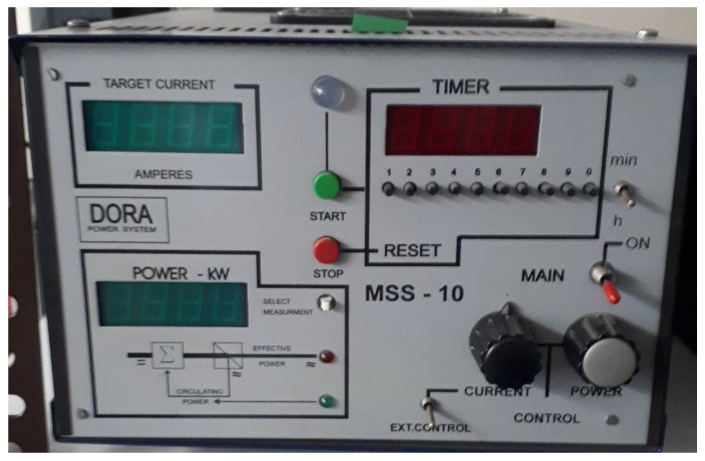
Impulse electric power supply.

**Figure 5:**
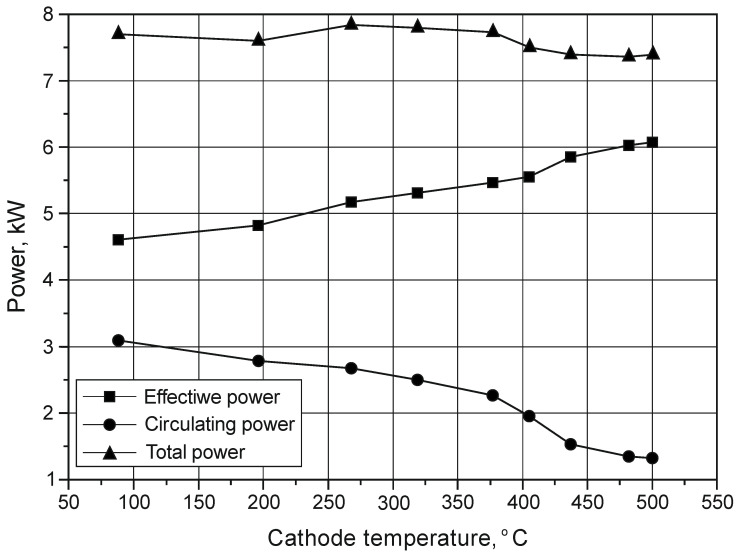
The influence of cathode temperature on the efficiency of using the total power supplied by the electric power supply during the nitriding process of Grade 5 titanium alloy.

From the point of view of the correctness and efficiency of the nitriding process, the effective power—being part of the total power—is of particular importance. It is transferred to increase the energy of ions in the glow discharge process. The rest of the power (the so-called circulating power) is returned to the power supply. Measurements of power provided to the device for glow processing were performed in constant 15 min time intervals. Values of the total power, effective power and circulating power were read from an impulse electric power supply, i.e., Dora Power System MSS-10 (Dora, Wroclaw, Poland) (Figure 4). The total power provided to the device during the process was within the range 7.4 to 7.8 kW. The results of the authors’ own tests allowed them to conclude that the degree of power use of the power supply depends on the cathode temperature (Figure 5). It was found that, with increasing cathode temperature, the value of the circulating power decreases, while the effective power increases. It is combined with an increase in the degree of gas ionisation with increasing temperature.

Two variants of the position of the samples in the glow tube furnace chamber were used. In the first variant, the samples were placed directly on the cathode, while in the second variant, the samples placed on the cathode were additionally covered with an active screen supporting the nitriding process made of perforated titanium sheet with chemical composition close to the nitrided alloy (active screen method). The active screen was a basic cylinder without the bottom plane with dimensions: Φ 200 mm × 100 mm, with openings with a diameter of Φ 5 mm evenly distributed on its surface at a distance of 5 mm from each other. The surface of nitrided substrate under cathodic nitriding conditions is bombarded with ions with great energy value dependent on the value of the so-called cathode drop (approximately 100 V). The introduction of the active screen causes that in the area of the cathode drop, additional strong voltage impulses appear. They interact with the nitrogen ions present in this area. The duration of voltage impulses facilitates obtaining high speed values by the ions, which correspond to the value of kinetic energy of approximately 300 eV [26,27].

Nitrogen ions are implanted into the substrate material, forming a nonequilibrium zone saturated with nitrogen in its surface layer. The produced high nitrogen concentration gradient facilitates its diffusion deeply into the substrate material. Therefore, at the initial stage, diffusion occurs through the grain and then along the grain boundaries. The occurrence of different diffusion paths causes the formation of a nitrided layer with high homogeneity of the microstructure phase composition [26].

Similar phenomena occur also in the nitriding process without an active screen in the cathode drop area, in the near-surface area of the nitrided substrate. However, the concentration of ions present there is significantly lower than in the near-surface area of the substrate beyond the active screen due to the narrow width of this area [26].

The nitrogen diffusion depth was determined on the basis of elemental arrangement analysis on an optical emission spectrometer with glow discharge (GDS GD Profiler HR (HORIBA, Banten, Indonesia) with a Grimma discharge lamp with a 4 mm cathode diameter (IMN, Gliwice, Poland)).

Microhardness measurements of the nitrided layers were conducted using the Knoop method on a Future Tech FM7 (Future Tech Corp., Kawasaki, Japan) microhardness tester. Microhardness was measured using a 490.3 mN load. Observation of the microstructures was performed with an Axiovert microscope (Carl Zeiss, Oberkochen, Germany) with digital image recording.

**Figure 6:**
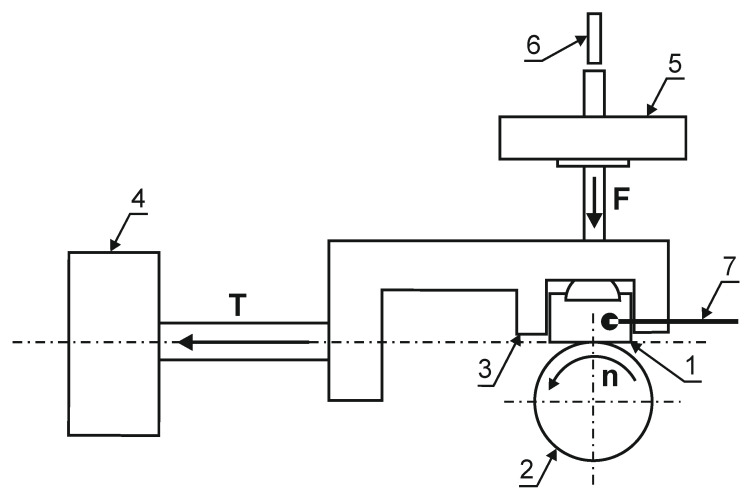
The diagram of a T-05 tester: (1) nitrided layer, (2) roll, (3) sample holder, (4) sensor for measuring the friction force, (5) load, (6) displacement sensor, (7) thermocouple.

To determine the tribological properties of the produced layers, the results of the abrasive wear resistance test and the values of the friction coefficient under dry friction conditions in the roller-block system were carried out on a T-05 tester (ITE, Radom, Poland) (Figure 6). The friction pair consisted of the nitrided surface of a cuboidal sample with dimensions of 4 × 8 × 12 mm and a roll with a diameter of 35 mm, made of 100Cr6 steel (ITE, Radom, Poland), with hardness of 62 HRC. During the test, the following parameters were recorded: friction force, displacement of the friction pair and sample temperature. In the test of abrasion resistance of nitrided layers on a Grade 5 titanium alloy substrate, due to their smaller depth in relation to, e.g., nitrided steels, the following were used: load 13.73 N, time 1.5 h (3 cycles, 0.5 h each), linear speed 1 m/s (friction path 3 × 1.8 km = 5.4 km).

## 3. Mechanism of Ion Nitriding Using the Active Screen Method

According to the conducted tests badaniami (X-ray phase analysis, GDEOS, microscopic tests, hardness tests, tests of linear distribution of elements’ concentration EDX), the ion nitriding process of Grade 5 titanium alloy consists of the following stages:

- production of titanium nitrides TiN
- decomposition of titanium nitride to lower titanium nitride and free nitrogen
  2TiN→Ti_2_N + N↓ (1)
- nitrogen diffusion into the substrate
- decomposition of lower titanium nitride to titanium and free nitrogen
  Ti2N →Ti + N↓(2)

The nitriding process of Grade 5 titanium alloy leads to the formation of zonal structure of surface layers—different depending on the variant of the nitriding process used. These zones are built from the nitrided surface from, accordingly: the zone of titanium nitrides (TiN and Ti2N), the zone of solid solution α (N) and the zone of the base material—the two-phase Ti α + β alloy. The introduction of the active screen especially increases the thickness of the Ti2N zone [28].

## 4. Results and Discussion

**Figure 7:**
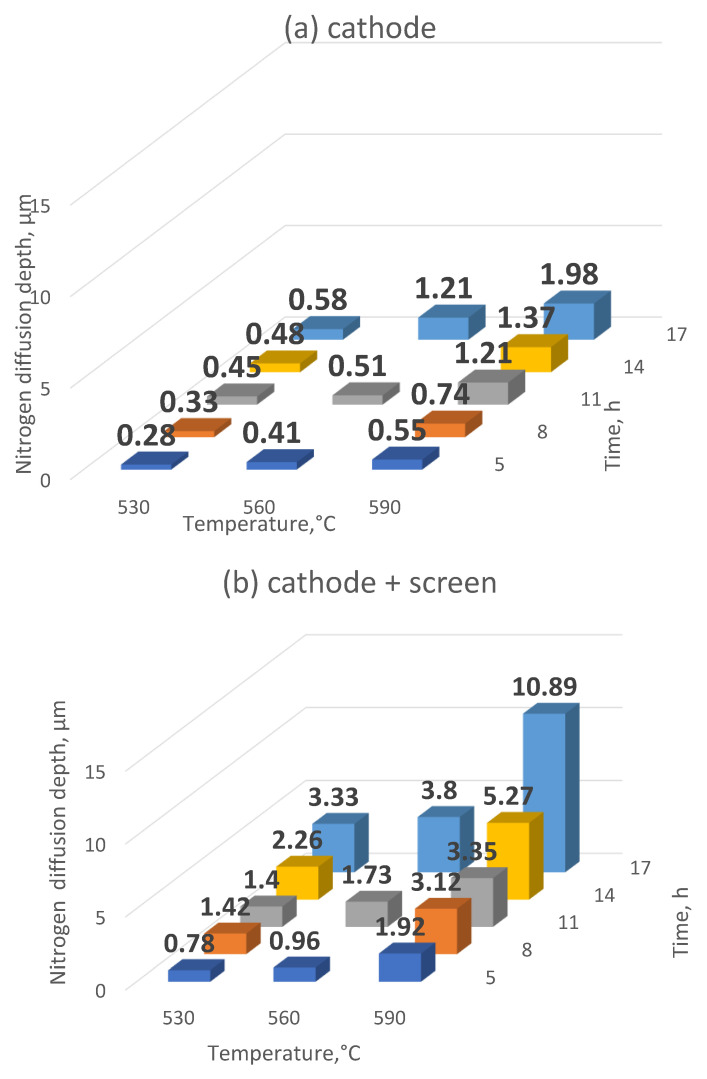
The depth of nitrided layers on the substrate of Grade 5 titanium alloy depending on the position of the samples in the glow chamber: (**a**) cathode, (**b**) cathode + screen.

**Figure 8:**
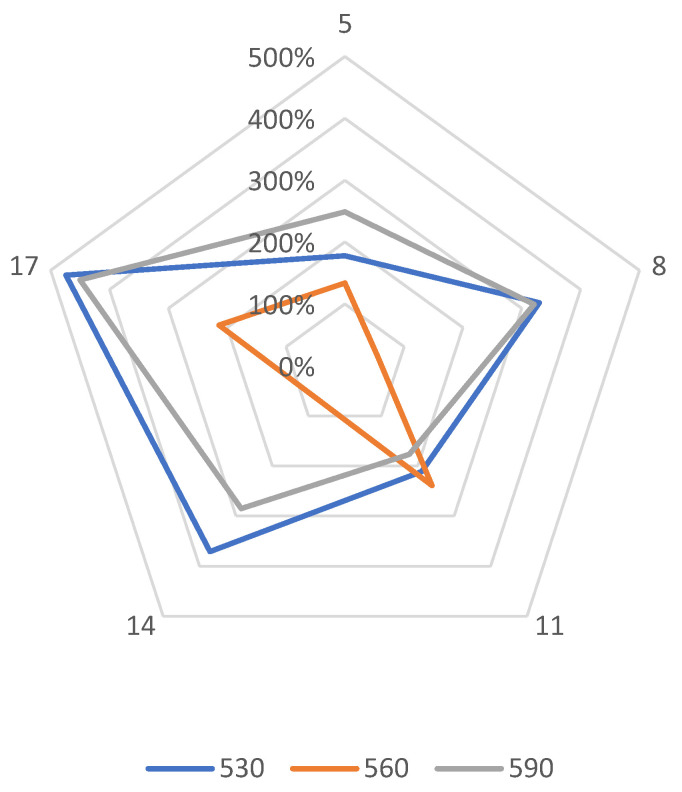
Percentage increase in the nitrogen diffusion depth of the material ion nitrided using the “active screen” method in relation to cathodic nitriding with different duration of the process (5 h, 8 h, 11 h, 14 h, 17 h).

The study of the depth of nitrogen diffusion deep into the nitrided substrate of Grade 5 titanium alloy, as the basic feature determining nitriding effectiveness, was used to determine the effectiveness of the ion nitriding process using the active screen method. It was found that increasing the temperature, as well as extending the duration of the nitriding process, results in an increase in the nitrogen diffusion depth in the Grade 5 titanium alloy selected for research (Figure 7). Applying the active screen method increases the intensity of the nitriding process because, depending on the parameters of the nitriding process using the active screen, a 2, 3—5, 7-fold increase in the thickness of the produced nitrided layers with respect to cathodic nitriding was found. It was found that the temperature increase, as well as the longer duration of the nitriding process, resulted in an increase in the nitrogen diffusion depth in the tested material. The increase in the nitrided layer under the active screen for cathodic nitriding was from 134% to 474% (Figure 8).

**Figure 9:**
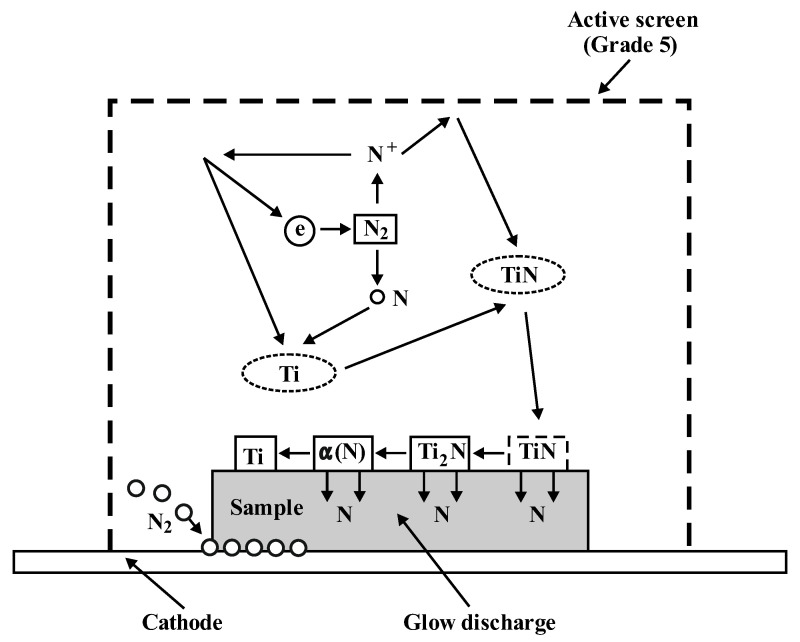
Model of the titanium nitriding process with the use of an active screen.

It can be assumed that the increase in the nitrogen diffusion depth is caused by a higher concentration of electrons inside the active screen, which increases the concentration of active plasma components in this area and the presence of molecular nitrogen that enables nitriding of the bottom of the sample directly adjacent to the cathode (Figure 9). In addition, the increase in the velocity of nitrogen ions that are implanted into the nitrided substrate leads to the creation of a non-equilibrium zone supersaturated with nitrogen, favouring a greater supply of nitrogen into the substrate. The above factors determine the higher growth rate of the nitrided layer in relation to cathodic nitriding [26]. Taking into account that the increase in temperature while maintaining a constant pressure will increase the free path of plasma active components, during the collisions of these components, they obtain greater kinetic energy, which contributes to greater plasma ionisation—and this affects the nitriding kinetics. It can therefore be concluded that, at higher temperatures, surface cathode sputtering is more effective.

**Figure 10:**
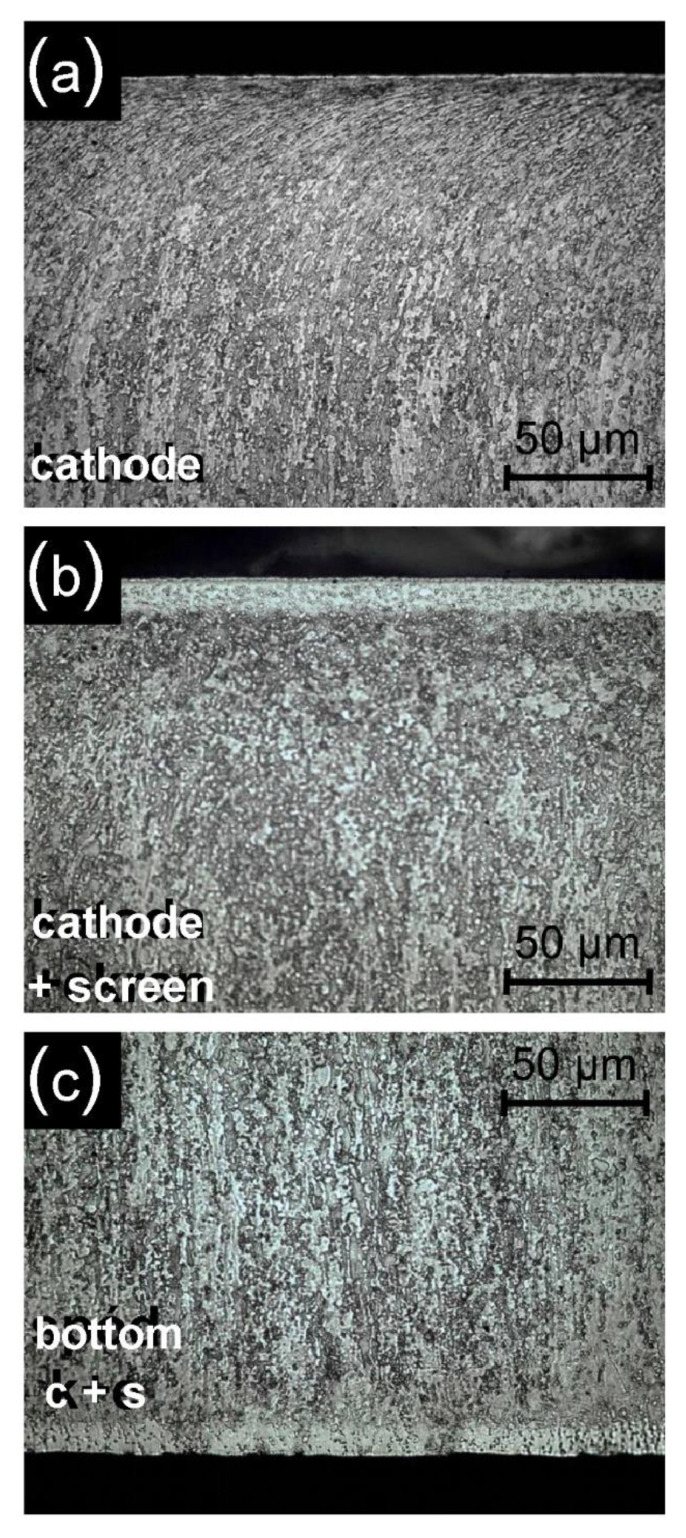
The microstructure of the nitrided layer on the substrate of Grade 5 titanium alloy for different variants of the samples’ distribution in the glow chamber: (**a**) cathode, (**b**) cathode + screen, (**c**) cathode + screen (with sample bottom). Process temperature T = 530 °C, time t = 17 h.

The observation of the microstructures obtained as a result of nitriding shows that the introduction of the active screen causes the formation of a dense zone of titanium nitrides on the substrate of Grade 5 titanium alloy, similarly as in the process of glow discharge nitriding on the cathode. It should be noted that with the adopted parameters of the nitriding process, the nitrided layer on Grade 5 titanium alloy is formed not only on surfaces surrounded by the glow discharge plasma (as is the case, for example, in metallic materials based on iron [27]), but also on the surface shielded from the glow discharge—on the surface adjacent to the cathode (Figure 10c). According to literature data, the nitride layer formed there at a significantly higher temperature over 700 °C [29]. As previously stated, the use of an active screen results in the production of nitrided layers of much greater thickness for cathodic nitriding in relation to nitriding (depending on time and temperature of the process, this increase ranges from 2.3 to 5.7) (Figure 10a,b) [30]. Microstructure tests (Figure 10) showed that, under the layer of nitrides formed, there is an area where grains of the base material deformed during machining have recrystallised (Figure 10a). The microstructure of the near-surface layer is characterised by strongly deformed grains and a local increase in hardness.

Measurements of the hardness of the nitrided layers on the substrate of Grade 5 titanium alloy showed that each ion nitriding process increased the hardness in relation to the hardness of this alloy in its initial state. The use of an active screen made it possible to increase the hardness several times in relation to cathodic nitriding (e.g., for the 17 h/530 °C process, the hardness at the load of 0.025 G for the samples nitrided on the cathode was 558 HK0.025, while the use of the additional active screen increased the hardness to 1021 HK0.025, with the raw material hardness being 346 HK0.025).

The abrasive wear resistance results correlate with the obtained hardness results. It should be emphasised that the applied method and conditions of the abrasion resistance test (roll-block) of nitrided layers formed on the substrate of the tested titanium alloy are difficult due to their small depth (of the order of a few μm). Nevertheless, the analysis of the obtained results shows that the nitrided layers, created for the adopted process conditions, significantly improve the abrasive wear resistance, additionally intensified by increasing the temperature and increasing the nitriding time of the tested alloy. The greatest increase in titanium abrasion resistance was found for surfaces after cathodic nitriding with an active screen. For example, for the 17 h/530 °C process, the weight loss for the cathode nitrided sample is 6.1 mg, and for the nitrided sample with the use of an active screen is 0.09 mg. The weight loss for the material in its initial state is as much as 23.8 mg. The highest, over a 260-fold increase in abrasive wear resistance was obtained for the layers formed during nitriding with the use of an active screen. For the cathodically nitrided layers, the increase was almost 4-fold in relation to the material before nitriding.

**Figure 11:**
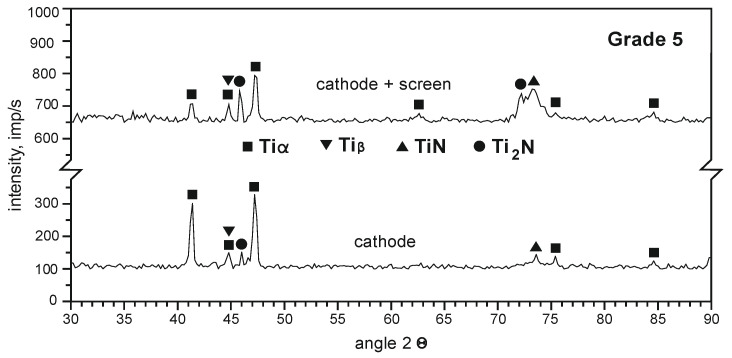
Diffractograms of the layer nitrided on the substrate of Grade 5 titanium alloy after various variants of ion nitriding, process temperature T = 530 °C, process time t = 17 h.

Based on the analysis of the phase composition of the layers formed on the substrate of Grade 5 titanium alloy, it can be stated that TiN and Ti_2_N nitrides are formed on the surface of the samples nitrided ionically on the cathode. The introduction of an active screen during nitriding on the cathode increases the nitride layer growth rate (a large number of intense TiN and Ti_2_N reflections occurs, Figure 11).

The authors of the paper found that nitrided layers are characterised by a high degree of surface development and the average height of the maximum unevenness on their surface—parameter Ra—depends on the conditions of the glow discharge nitriding process, especially temperature. Ions interacting with the surface of the nitrided substrate, depending on the position of the nitrided element in relation to the cathode and the area of the glow discharge, have different energy values. This results in a diversified degree of development of the substrate surface and is of great importance for the usable properties. For example, surface development is often important from the point of view of the biocompatibility of implants with the human body. Grade 5 titanium alloy is one of the basic metallic materials used for medical implants. The authors of the paper found that the surface topography, in addition to the chemical composition of the layer, has a large effect on the adhesion and growth rate of human fibroblasts to the surface of implants [31].

**Figure 12:**
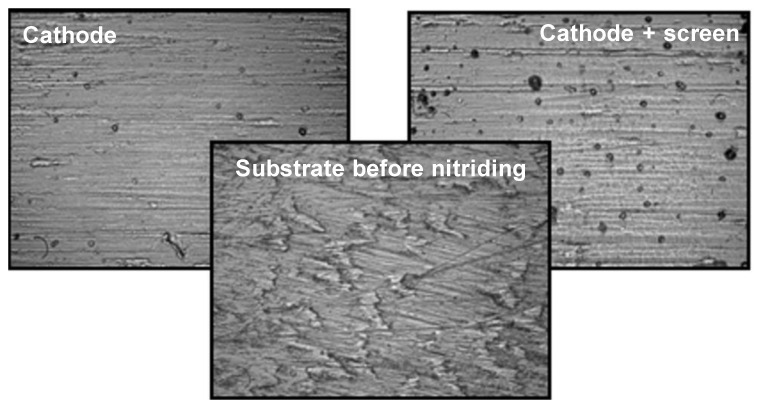
Microphotographs of the surface condition of Grade 5 titanium before and after nitriding in various areas of the glow discharge.

To determine how the applied variants of nitriding affect the development of the nitrided substrate, the authors of this study conducted tests, using a MultiMode V-Veeco Instruments (Bruker, Billerica, MA, USA) atomic force microscope, on the roughness of Grade 5 titanium alloy after nitriding. Each of the applied nitriding variants resulted in surface development, which is manifested by an increase in roughness from the value of 0.78 nm for the initial state (before the nitriding process) to the value of 4.43 nm for the active screen nitriding method, and 5.51 nm for the cathode nitriding variant (for the process variant: time 17 h, temp. 530) (Figure 12, Table 3).

## 5. Conclusions

The conducted tests and analysis of the results show that the use of an active screen in the ion nitriding process of two-phase Grade 5 titanium alloy increases the intensity of the nitriding process, as well as increases the temperature of this process.

The use of an active screen causes additional voltage pulses under this screen, the values of which are several times higher than during cathodic nitriding. These pulses cause the ions and other active components of the plasma to gain high speed, as a result of which the active components of the plasma are introduced into the substrate material, creating a zone supersaturated with nitrogen, which facilitates the diffusion of nitrogen deep into the substrate.

The increase in temperature resulting from the use of an active screen contributes to the formation of a nitrided layer, not only on the surface in the plasma environment, but also on the surface adjacent to the cathode, which proves the presence of molecular nitrogen.

On the basis of the obtained results, it can be concluded that both the increase in the process temperature and the duration of the process result in an increase in the nitrogen diffusion depth into the substrate in Grade 5 titanium alloy. The use of an active screen contributes to a further increase in the intensity of the nitriding process, which is reflected in the increase in the thickness of the nitrided layer.

The conducted tests on the observation of the microstructure allowed the authors to identify the area under the layer of nitrides, in which the grains deformed during the machining of the substrate underwent recrystallisation. The use of an active screen and the resulting increase in temperature contributed to the further growth of previously recrystallised grains.

The analysis of the results of tests on the microstructure and phase composition of nitrided layers on the substrate of two-phase titanium alloys was the basis for the development of models of their structure after unconventional methods of glow discharge nitriding. The microstructure of these layers consists of titanium nitrides TiN, Ti_2_N and the α (N) phase.

One of the significant benefits of using an active screen in the nitriding process is the significant increase in hardness compared to the conventional nitriding process (Table 3).

A similar increase, although characterised by a much greater intensity, was observed for the abrasion. (In relation to the starting material: a 4-fold increase was recorded after conventional nitriding, and a 260-fold increase after the active screen was used).

Grade 5 titanium alloy is one of the basic metallic materials used for medical implants. For this type of products, the appropriate degree of surface development, which affects the adhesion and growth rate of human fibroblasts, is important (apart from the chemical composition of the layer). The variants of the nitriding process developed by the authors contribute to a significant increase in the actual surface area of Grade 5 titanium alloy, which is the result of an intense interaction of ions with the substrate surface.

## Figures and Tables

**Table 1:** Chemical composition of Grade 5 titanium alloy/wt. %.

C	Fe	O	N	H	Al	V	Ti
0.02	0.38	0.11	0.016	0.0026	6.4	4.5	Rest

**Table 2:** Parameters of nitriding processes of Grade 5 titanium alloy.

Process	Temperature, °C	Time, h	Pressure, Pa	Chemical Composition of the Working Atmosphere
1	590	14	150	H_2_ 25% + N_2_ 75%
2	590	8		
3	560	5		
4	530	8		
5	530	14		
6	560	17		
7, 8, 9	560	11		

**Table 3:** Summary of roughness, hardness and wear resistance results.

**Surface Roughness, nm**		
Before nitriding	Cathode	Cathode + Screen
0.78	5.51	4.43
**Hardness 0.025/0.05 HK**		
Before nitriding	Cathode	Cathode + Screen
346/337	558/478	1021/767
**Abrasive Wear Resistance—Weight Loss, mg**		
Before nitriding	Cathode	Cathode + Screen
23.8	6.1	0.09

## Data Availability

The data underlying this article will be shared on reasonable request from the corresponding author.
